# MIMIC: a Python package for simulating, inferring, and predicting microbial community interactions and dynamics

**DOI:** 10.1093/bioinformatics/btaf174

**Published:** 2025-05-23

**Authors:** Pedro Fontanarrosa, Chania Clare, Alex J H Fedorec, Chris P Barnes

**Affiliations:** Research Department of Cell and Developmental Biology, University College London, London, WC1E 6BT, United Kingdom; Research Department of Cell and Developmental Biology, University College London, London, WC1E 6BT, United Kingdom; Research Department of Cell and Developmental Biology, University College London, London, WC1E 6BT, United Kingdom; Research Department of Cell and Developmental Biology, University College London, London, WC1E 6BT, United Kingdom

## Abstract

**Summary:**

The study of microbial communities is vital for understanding their impact on environmental, health, and technological domains. The Modelling and Inference of MICrobiomes Project (MIMIC) introduces a Python package designed to advance the simulation, inference, and prediction of microbial community interactions and dynamics. Addressing the complex nature of microbial ecosystems, MIMIC integrates a suite of mathematical models, including previously used approaches such as Generalized Lotka-Volterra (gLV), Gaussian Processes (GP), and Vector Autoregression (VAR) plus newly developed models for integrating multi-omic data, to offer a versatile framework for analyzing microbial dynamics. By leveraging Bayesian inference and machine learning techniques, MIMIC provides the ability to infer the dynamics of microbial communities from empirical data, facilitating a deeper understanding of their complex biological processes, unveiling possible unknown ecological interactions, and enabling the design of microbial communities. Such insights could help to advance microbial ecology research, optimizing biotechnological applications, and contribute to environmental sustainability and public health strategies. MIMIC is designed for flexibility and ease of use, aiming to support researchers and practitioners in microbial ecology and microbiome research.

**Availability and implementation:**

MIMIC is freely available under the MIT License at https://github.com/ucl-cssb/MIMIC. It is implemented in Python (version 3.7 or higher) and is compatible with Windows, macOS, and Linux operating systems. MIMIC depends on standard Python libraries including NumPy, SciPy, and PyMC. Comprehensive examples and tutorials (including the main text demonstrations) are provided as Jupyter notebooks in the examples/directory and at the MIMIC Docs website, along with detailed installation instructions and real-world data use cases. The software will remain freely available for at least two years following publication. A code snapshot for this publication is also available at Zenodo: https://doi.org/10.5281/zenodo.15149003.

## 1 Introduction

Microbial communities play a critical role in maintaining ecosystem functions, influencing human health (including digestion, immunity, and mental health), and impacting environmental processes such as nutrient cycling and pollution degradation. There is a potential to engineer microbial communities for specific therapeutic and environmental purposes and functions. The gut microbiota is a dynamic community composed of a variety of microbes, metabolites, and environmental perturbations, with complex direct and indirect interactions between all components. When balanced, this network can support healthy gut function and protect against infection (Reissbrodt *et al.* 2009, Cryan and O’Mahony 2011, Deriu *et al.* 2013), but in dysbiosis it can contribute to a multitude of health problems, such as colorectal cancer (Fan *et al.* 2021), diabetes (Wang *et al.* 2012), cardiovascular disease (Koeth *et al.* 2013), and obesity (Bäckhed *et al.* 2004). Within this community, each species has distinct nutrient preferences and colonization strategies, which result in complex intra-species dynamics that are constantly subjected to a changing environment. The nature of these dynamics also changes over time (Caporaso *et al.* 2011), where some species can contribute to healthy microbiota until an opportunity arises and they become pathogenic and harmful to human health (Kamada *et al.* 2013). In order to understand and control this complex and crucial community, it is important to untangle the nature of these interactions.

Mathematical models are essential tools for unraveling the complexities of biological systems, providing insights that can drive both understanding and innovation. These models can help us not only understand interactions but also design them with specific functions (robustness, permanence, etc.) (Gonze *et al.* 2018, Karkaria *et al.* 2021). Simpler models, like *Generalized Lotka-Volterra* (gLV), *Consumer Resource* (CR), and *Vector Autoregression* (VAR), can effectively predict community dynamics and offer manageable frameworks for studying microbial interactions (Stein *et al.* 2013). However, the complexity of some biological systems can pose significant challenges, particularly when parameters and structures are poorly understood. The experimental data needed to infer these models are often extensive, multidimensional, and, in most cases, not sufficiently comprehensive or high-quality to support accurate model inference, necessitating the use of advanced computational approaches to compensate for these limitations (Cao *et al.* 2017).

Machine learning and Bayesian inference have emerged as powerful tools to address these challenges. Machine learning can handle complex multidimensional data, extract meaningful features, infer model structures and parameter values, and integrate diverse modelling methods with high accuracy (Reel *et al.* 2021, Sequeiros *et al.* 2023). However, despite their effectiveness, these methods can sometimes produce models that are difficult to interpret biologically, which may limit their utility in the engineering of biological systems (Chen *et al.* 2024).

Designing microbial communities for specific purposes is a challenging task due to the inherent complexity of biological systems. While mathematical models provide valuable insights, they often require simplifications that limit their accuracy. Machine learning, on the other hand, excels at handling complex data but can lead to over-fitting and lack of interpretability.

To address these challenges, we introduce the *Modelling and Inference of MICrobiomes Project* (MIMIC). MIMIC is an open-source Python package designed to integrate simulation, inference, and prediction of microbial community interactions and dynamics. It combines simplistic mechanistic models with machine learning approaches, creating interpretable model structures and accurately inferring parameters. MIMIC enhances mathematical inference, enabling better models to simulate and design stable bacterial communities to facilitate the study of microbial community interactions and dynamics.

## 2 Implementation and features

MIMIC is a Python package for simulating microbial ecosystems, inferring interactions from data, and predicting community dynamics. It is designed to complement, rather than replace, existing modeling and inference software such as MICOM (Diener *et al.* 2020), COBRA (Heirendt *et al.* 2019), and MDSINE2 (Bucci *et al.* 2016, Gibson and Gerber 2018). Unlike MICOM, which uses constraint-based metabolic modeling, MIMIC focuses on inferring microbial interactions from time-series data using Bayesian inference and dynamic modeling approaches. Compared to MDSINE2, MIMIC supports a broader range of modeling frameworks (gLV, VAR, and CR models) and uniquely enables species-metabolite interaction inference, a feature not typically available in MICOM or MDSINE2. Additionally, MIMIC integrates Bayesian and linear inference methods to provide uncertainty quantification in parameter estimation, enhancing its versatility for microbiome research. These capabilities make MIMIC particularly well-suited for longitudinal microbial datasets, multi-omics integration, and ecological interaction inference. A detailed comparison of MIMIC and existing tools can be found in the Supplementary Table S1.

**Figure 1.**
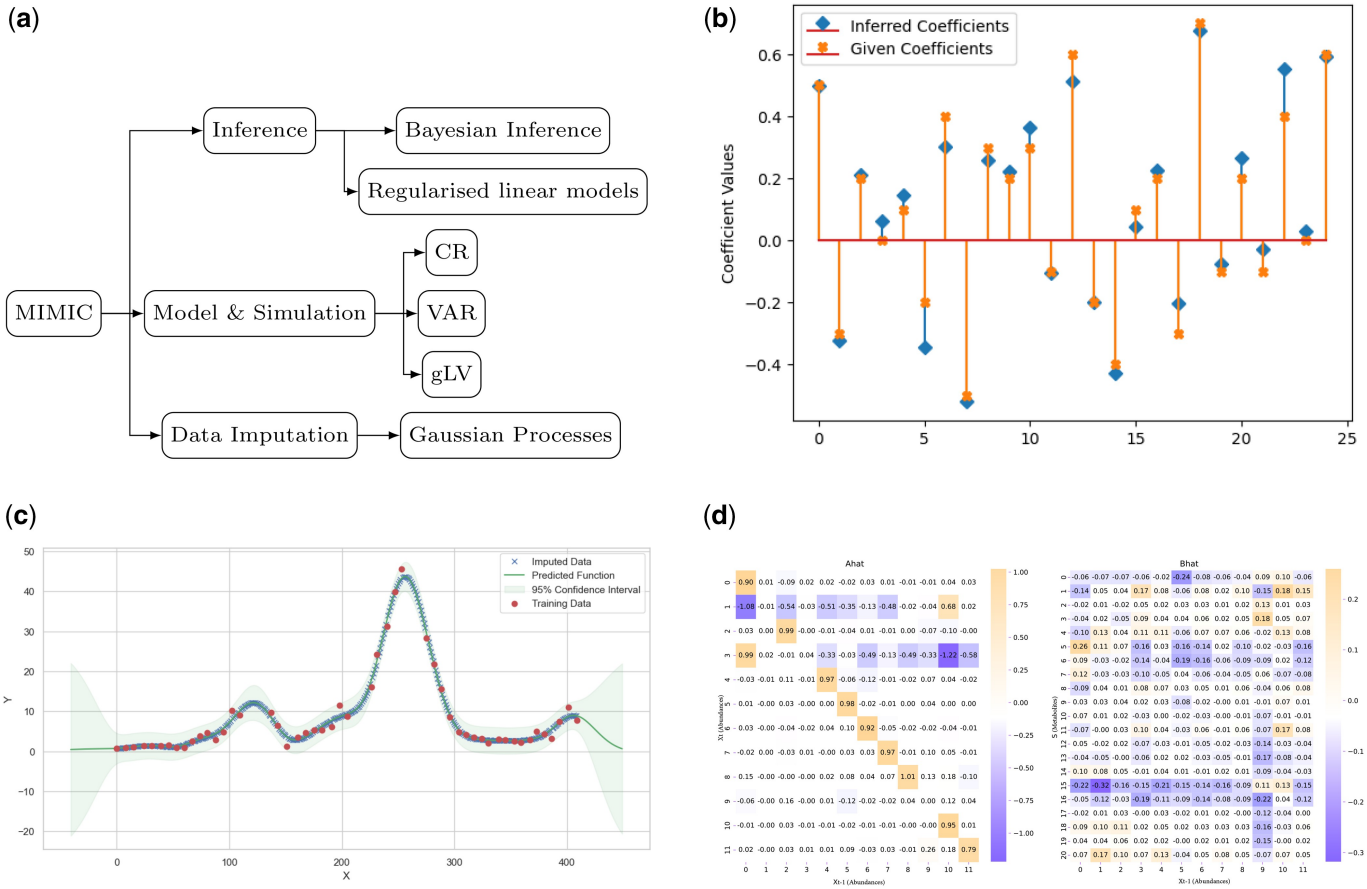
Overview of MIMIC’s capabilities and an example inference workflow. (a) A schematic summarizing MIMIC’s core components: *Data Imputation* (Gaussian Processes), *Model & Simulation* (gLV, VAR, CR), and *Inference* (Bayesian and linear approaches). (b) Comparison of the original (“given”) interaction coefficients used to simulate a synthetic VAR dataset versus the coefficients inferred by MIMIC’s Bayesian approach. (c) An example of Gaussian Process regression for imputing missing time-series data in microbial abundance measurements; the solid line is the mean prediction, shaded areas indicate confidence intervals, and dots are observed data points. (d) Heatmaps of the inferred $\hat{A}$ and $\hat{B}$ matrices from a real-world wastewater treatment dataset using MVAR inference, indicating how changes in microbial abundances ($\hat{A}$) influence current abundances and how changes in abundances affect metabolite concentrations ($\hat{B}$). High-intensity squares suggest stronger interaction effects. Together, these panels illustrate how MIMIC can (i) simulate data under various models, (ii) impute missing observations, and (iii) infer interaction networks from both synthetic and real-world datasets.

Designed as a modular and open-source framework, MIMIC facilitates collaborative development, allowing researchers to extend and integrate it with other computational tools and libraries. An overview of MIMIC’s structure and key functionalities is shown in Fig. 1a.

### 2.1 Data imputation

MIMIC implements a Gaussian Process regression for time-series data imputation, providing probabilistic predictions with confidence intervals. This approach is particularly useful in microbial community datasets that often suffer from incomplete observations.

### 2.2 Model and simulation

MIMIC uses commonly used models for simulation and analysis in microbial community research such as the gLV, CR, and VAR models which have been shown to effectively predict community dynamics and offer manageable frameworks and valuable insights into microbial community dynamics (Stein *et al.* 2013, Bucci *et al.* 2016, Gibbons *et al.* 2017, Hannaford *et al.* 2023).

### 2.3 Inference algorithms

MIMIC implements inference methods for parameter estimation using both Bayesian and linear approaches. In its Bayesian workflow, MIMIC relies on the PyMC library (Patil *et al.* 2010) for posterior sampling and uncertainty quantification. When users do not supply their own priors, the package automatically assigns sensible defaults: Normal priors for interaction coefficients and intercepts, Horseshoe priors to promote sparsity, and an LKJ prior for covariance matrices (Carvalho *et al.* 2009, Hannaford *et al.* 2023).

## 3 Results

We demonstrate MIMIC’s capabilities using both synthetic and real-world datasets. First, we simulated microbial abundances using the VAR model with specific interaction parameters, then used Bayesian inference to recover these parameters. Second, we applied MIMIC to a real-world dataset of microbial abundances and metabolite concentrations from a wastewater treatment facility (Herold *et al.* 2020). We chose the VAR model for this example because it conveniently handles multiple species and metabolite data without requiring extensive mechanistic assumptions, illustrating MIMIC’s Bayesian inference capabilities. However, MIMIC fully supports other methods and models (including gLV, CR, and MVAR), demonstrated with different datasets in our GitHub repository.

Selecting the optimal modeling framework depends on both the biological context and the nature of the available data. For instance, the gLV model is well suited for systems where pairwise interactions dominate and linear approximations of species interactions are reasonable—often the case in controlled *in vitro* experiments with a limited number of species. In contrast, CR models explicitly incorporate resource dynamics, making them preferable when nutrient availability, resource competition, or metabolic cross-feeding are expected to play a central role in community dynamics. Meanwhile, VAR models provide a flexible statistical approach that leverages high-resolution time-series data to capture lagged relationships among multiple species (and metabolites) without imposing strict mechanistic assumptions. Ultimately, your choice should align with your research question, the system’s complexity, and the data resolution; model fit comparisons using criteria like *Bayesian Information Criterion* (BIC) or *Akaike Information Criterion* (AIC) can further guide the selection. Importantly, MIMIC is designed to support all these frameworks (and more in the future), enabling users to systematically compare and select the model that best captures the underlying ecological processes.

### 3.1 Simulation and inference of synthetic data

This section demonstrates how MIMIC can simulate microbial abundance time-series data using the VAR method and subsequently infer the interaction coefficient matrix from the simulated data.

The MIMIC package can simulate data from a *Vector Autoregression* (VAR) process [Equation (1)], where each variable’s current value is influenced by the immediately preceding values of all variables in the system. The general formula for a VAR process with n variables is given by

(1) $$X_{t}=A\cdot X_{t-1}+\epsilon_{t},$$

where $X_{t}$ is the vector of variables at time t, A is the matrix of coefficients capturing the influence of each variable’s previous value on the current value of all variables in the system, and $\epsilon_{t}$ is the vector of error terms. We assumed the error to be normally distributed with mean 0 and standard deviation 1.5.

Using MIMIC’s Bayesian inference for VAR processes, we recovered the coefficient matrix from the simulated data. The inferred parameters closely matched the ground truth, validating the method (Fig. 1b).

### 3.2 Case study

We applied MIMIC’s data imputation and inference methods to a real-world dataset from a wastewater treatment plant provided by Herold *et al.* (2020). This dataset includes meta-proteomic data for metabolites and meta-genomic data for microbial abundances.

First, we used MIMIC’s Gaussian Process regression to impute missing data (Fig. 1c). After imputation, we applied a VAR model to infer the relationships between microbial abundances and metabolite concentrations:

(2) $$X_{t}=A\Delta X_{t-1}+\epsilon_{x},$$

 

(3) $$S_{t}=B\Delta X_{t-1}+\epsilon_{y},$$

where $\Delta X_{t}$ is the change in microbial abundances, A represents the influence of past abundances on current abundances, $S_{t}$ is the metabolite concentrations, B represents the influence of microbial changes on metabolites, and $\epsilon_{x}$, $\epsilon_{y}$ are error terms. In this study, we performed inference using relative abundances due to the nature of the available data.

After using the VAR inference methods from MIMIC, we obtained the results shown in Fig. 1d. In the example presented here, MIMIC sets up a Bayesian model for the interaction matrices (A and B) and noise terms in PyMC, then uses Markov Chain Monte Carlo (MCMC) to sample from the posterior distributions, providing both point estimates and credible intervals for all parameters. Since custom priors were not specified, we used MIMIC’s default Normal priors for interaction coefficients and intercepts, along with an LKJ prior for covariance.

The heatmaps show the results of the VAR model inference using MIMIC, with two matrices $\hat{A}$ and $\hat{B}$:

-
$\hat{A}$ **Matrix (Left Heatmap)**: Represents the coefficients capturing the influence of past microbial abundances on current microbial abundances. Strong diagonal elements indicate that each species’ past abundance strongly influences its current abundance. Off-diagonal elements suggest weaker cross-species interactions, with some significant competitive or facilitative relationships.
-
$\hat{B}$ **Matrix (Right Heatmap)**: Represents the coefficients capturing the influence of changes in microbial abundances on metabolite concentrations. The diverse range of interactions suggests complex influences where some microbial species promote while others inhibit the production or consumption of specific metabolites.

To further assess the reliability of the inferred interaction matrices, we conducted a **stability analysis** on the median *species-species interaction matrix* ($A_{h}$). Stability was evaluated by computing the eigenvalues of the median $A_{h}$ matrix, with stability defined as all eigenvalues having an absolute value <1. Our results confirm that the **median inferred**  $A_{h}$  **matrix is stable**.

Additionally, we assessed the stability of all posterior samples, computing the percentage of sampled matrices that satisfy the stability criterion. We found that **87.48% of the sampled matrices were stable**. This suggests that while the majority of inferred interaction structures are stable, a subset of samples exhibit instability, potentially indicating uncertainty in specific inferred interactions. The full breakdown of this analysis is provided in Supplementary Tables S2 and S3.

While these results primarily demonstrate MIMIC’s technical capabilities, inferred parameters and interaction matrices can highlight which microbial species or metabolites most strongly promote or inhibit others. Such insights help identify potential keystone species or metabolic drivers, guide experimental validation, and inform targeted interventions—such as dietary changes or probiotic strategies—that encourage beneficial communities and suppress harmful ones. For deeper insights and interpretations of the example shown here, please visit our GitHub repository.

### 3.3 Additional methods and examples

Due to space constraints, additional examples demonstrating the gLV, CR, and MVAR models (along with real-world datasets) can be found in the GitHub repository at https://github.com/ucl-cssb/MIMIC/tree/master/examples and documented in detail at our documentation site https://ucl-cssb.github.io/MIMIC/.

## 4 Discussion and future directions

MIMIC provides a robust and versatile tool for modelling microbial community interactions and dynamics. By leveraging advanced Bayesian inference and sparse modelling techniques, MIMIC can identify significant interactions and predict community dynamics with high reliability. While dynamic models such as the gLV and VAR frameworks have proven valuable for simulating and inferring microbial interactions in controlled *in vitro* or synthetic communities—where high-frequency sampling and relatively simple pairwise interactions can be assumed—they face significant challenges in more complex ecosystems such as the human gut microbiome (Momeni *et al.* 2017, Carr *et al.* 2019, Dedrick *et al.* 2023). In these environments, the intrinsic growth rates of many microbes often occur on timescales that are much shorter than those captured by typical sampling frequencies, and many interactions are mediated by diffusible chemicals or involve higher-order, nonadditive effects that violate the core assumptions of pairwise models. Furthermore, if the recorded abundances are relative and not absolute, it is not possible to ensure global identifiability (Remien *et al.* 2021). However, in other contexts—such as when high-resolution time-series data are available or when researchers seek to elucidate potential metabolic drivers—MIMIC not only reveals the underlying interaction structure but also helps determine the metabolic influences that govern microbial dynamics.

Several tools exist for modeling microbial dynamics, such as MDSINE (Bucci *et al.* 2016), MICOM (Diener *et al.* 2020), and MDITRE (Maringanti *et al.* 2022). However, MIMIC aims to complement rather than replace these existing tools by introducing new modeling approaches and inference methods not currently implemented in these packages. While MDSINE focuses on time-series analysis and MICOM on genome-scale metabolic models, MIMIC provides methods to integrate longitudinal data, providing a versatile toolset for simulating, inferring, and predicting microbial community interactions. It supports a broader range of model types, including VAR and Gaussian Processes, alongside machine learning and Bayesian inference methods. Importantly, MIMIC serves as a foundational platform for bringing together disparate modelling methods under one roof, enabling researchers to easily integrate and leverage techniques developed by others. This ethos is in line with the vision of open collaborative science, as highlighted in recent discussions on collaborative research frameworks (Ghosh *et al.* 2024). By promoting interoperability and the integration of various modelling tools, MIMIC aims to contribute to a more robust and versatile ecosystem for microbial research, encouraging contributions from a diverse community of researchers. This approach not only enhances the analytical capabilities available to scientists but also supports reproducibility and the advancement of the field as a whole.

Future work will focus on implementing more machine learning methods coupled with mechanistic models to obtain inferred model structures and parameters that are biologically interpretable (Yuan and Shou 2022). Specifically, we plan to incorporate techniques such as Gaussian processes and physics-informed neural networks for modelling and inferring nonlinear systems. These enhancements will provide powerful tools for both understanding and engineering microbial communities for specific functions.

## Supplementary Material

btaf174_Supplementary_Data

## Data Availability

The data underlying this article are available in Zenodo, at https://doi.org/10.5281/zenodo.15149003.
