# Supplementary material for: MIMIC: a Python package for simulating, inferring, and predicting microbial community interactions and dynamics
Source: Bioinformatics. 2025 May 23;41(5):btaf174. doi: 10.1093/bioinformatics/btaf174 (PMC12119135; doi:10.1093/bioinformatics/btaf174)
Supplement: btaf174_Supplementary_Data [file btaf174_supplementary_data.pdf]

Supplemental Data for "MIMIC: A Python Package for Simulating, Inferring, and Predicting Microbial Community Interactions and Dynamics" Application Note

Pedro Fontanarrosa*[1], Chania Clare[1], Alex J.H. Fedorec[1], and Chris P. Barnes[1]

[1]Cell and Developmental Biology Dept., University College London, London, UK

April 11, 2025

*Corresponding author: pfontanarrosa@gmail.com

Correlation Coefficients $\hat{r}$ for Simulated vs. Inferred Parameters in VAR Model Inference

References

Bucci, Vanni et al. (June 2016). "MDSINE: Microbial Dynamical Systems INference Engine for Microbiome Time-Series Analyses". In: *Genome Biology* 17.1, p. 121. ISSN: 1474-760X. DOI: 10.1186/s13059-016-0980-6. (Visited on 05/30/2024).

Diener, Christian, Sean M. Gibbons, and Osbaldo Resendis-Antonio (Jan. 2020). "MICOM: Metagenome-Scale Modeling To Infer Metabolic Interactions in the Gut Microbiota". In: *mSystems* 5.1, 10.1128/msystems.00606–19. DOI: 10.1128/msystems.00606-19. (Visited on 01/02/2024).

Gibson, Travis and Georg Gerber (Oct. 2018). "Robust and Scalable Models of Microbiome Dynamics". In: *Proceedings of the 35th International Conference on Machine Learning*. Ed. by Jennifer Dy and Andreas Krause. Vol. 80. Proceedings of Machine Learning Research. PMLR, pp. 1763–1772.

Heirendt, Laurent et al. (2019). "Creation and analysis of biochemical constraint-based models using the COBRA Toolbox v. 3.0". In: *Nature protocols* 14.3, pp. 639–702.

Table S1: Comparison of MIMIC, MICOM (Diener, Gibbons, and Resendis-Antonio 2020), MDSINE2 (Bucci et al. 2016; Gibson and Gerber 2018), and COBRA Toolbox (Heirendt et al. 2019)

Feature	MIMIC	MICOM	MDSINE2	COBRA Toolbox
Programming Language	Python	Python	Python	MATLAB (with Python bindings)
Modeling Frameworks	gLV, VAR, Consumer Resource (CR)	Flux Balance Analysis (FBA)	Generalized Lotka-Volterra (gLV)	Constraint-based metabolic modeling
Inference Methods	Bayesian (PyMC), Linear Regression	L2 Regularization	Fully Bayesian (MCMC, Markov Chain Monte Carlo)	Linear Optimization (LP, MILP)
Species-Species Interaction	Yes	No	Yes	No
Species-Metabolite Interaction	Yes (e.g., MVAR model)	Yes (via metabolic constraints)	No	Yes (metabolic flux analysis)
Time-Series Data Analysis	Yes	No (steady-state only)	Yes	No (steady-state only)
Uncertainty Quantification	Yes (via PyMC Bayesian inference)	No	Yes (Bayesian posterior inference)	No
Metabolic Modeling	No	Yes (metabolic modeling)	No	Yes (metabolic modeling)
Ease of Use	High (modular Python framework, Jupyter tutorials)	Moderate (Python-based but requires COBRApy knowledge)	Moderate (computationally intensive, requires Bayesian modeling)	Low (requires MATLAB and metabolic knowledge)
Open-Source	Yes (MIT License, GitHub)	Yes (Open-source, GitHub)	Yes (GNU GPL License, GitHub)	Yes (Open-source, GitHub)

Table S2: R-hat Values for Species-Species Interactions (Ah Matrix)

Species<br>Species	0	1	2	3	4	5	6	7	8	9	10	11
0	1.39	1.16	1.12	1.23	1.10	1.34	1.47	1.13	1.15	1.11	1.42	1.15
1	1.19	1.13	1.40	1.23	1.22	1.47	1.56	1.52	1.36	1.25	1.72	1.60
2	1.26	1.32	1.46	1.42	1.17	1.07	1.46	1.26	1.48	1.46	1.12	1.09
3	1.37	1.23	1.40	1.25	1.34	1.62	1.38	1.42	1.47	1.09	1.25	1.15
4	1.44	1.42	1.44	1.33	1.25	1.13	1.13	1.11	1.31	1.12	1.16	1.27
5	1.40	1.48	1.21	1.44	1.09	1.40	1.39	1.23	1.43	1.07	1.09	1.11
6	1.19	1.25	1.13	1.09	1.25	1.32	1.42	1.08	1.21	1.45	1.13	1.15
7	1.50	1.12	1.13	1.20	1.11	1.52	1.41	1.28	1.27	1.23	1.26	1.11
8	1.35	1.12	1.17	1.09	1.13	1.11	1.22	1.45	1.09	1.10	1.33	1.14
9	1.43	1.28	1.42	1.28	1.13	1.20	1.45	1.20	1.51	1.23	1.47	1.10
10	1.10	1.12	1.13	1.08	1.46	1.48	1.32	1.13	1.09	1.47	1.40	1.45
11	1.42	1.11	1.31	1.13	1.47	1.45	1.10	1.54	1.18	1.33	1.14	1.46

Table S3: R-hat Values for Species-Metabolite Interactions (Bh Matrix)

Species<br>Matabolite	0	1	2	3	4	5	6	7	8	9	10	11
0	1.33	1.62	1.52	1.57	1.84	1.22	2.13	1.64	1.77	1.19	1.68	1.59
1	1.40	1.22	1.36	1.78	1.71	1.54	1.48	1.74	1.65	1.44	1.40	1.81
2	1.21	1.48	1.17	1.16	1.47	1.46	1.25	1.22	1.61	1.59	1.26	1.35
3	1.98	1.87	1.48	1.45	1.84	1.22	1.94	2.11	1.79	1.43	1.90	1.67
4	1.46	1.88	1.37	1.68	1.74	1.61	1.72	1.70	1.67	1.46	1.48	1.58
5	1.40	1.52	1.35	1.60	1.63	1.37	1.43	1.54	1.38	1.21	1.33	1.71
6	1.60	1.55	1.53	1.35	1.57	1.54	1.55	1.54	1.15	1.41	1.61	1.12
7	1.60	1.83	1.51	2.01	2.04	1.30	2.22	1.68	1.75	1.45	1.52	1.79
8	2.08	2.17	1.58	1.75	1.84	1.90	1.97	1.50	1.78	1.36	1.57	1.22
9	1.39	1.34	1.52	2.06	1.75	1.60	2.03	1.80	1.99	1.23	1.69	1.88
10	1.47	1.11	1.55	1.77	1.29	1.47	1.59	1.56	1.46	1.12	1.45	1.75
11	1.54	1.66	1.54	1.82	1.61	1.24	1.85	1.66	1.63	1.15	1.61	1.70
12	1.24	1.37	1.23	1.30	1.85	1.72	1.74	1.37	1.79	1.56	1.22	1.27
13	1.25	1.75	1.74	1.50	1.60	1.63	1.53	1.81	1.64	1.50	1.31	1.62
14	1.42	1.24	1.38	2.06	1.59	1.61	1.77	1.18	1.51	1.20	1.45	1.67
15	1.25	1.46	1.11	1.84	1.54	1.15	1.65	1.24	1.62	1.19	1.38	1.75
16	1.29	1.51	1.44	1.93	1.62	1.47	1.83	1.55	1.91	1.48	1.43	1.92
17	1.48	1.39	1.47	1.64	1.39	1.27	1.43	1.27	1.61	1.10	1.28	1.63
18	1.42	1.29	1.56	1.82	1.48	1.53	1.63	1.67	1.67	1.08	1.42	1.78
19	1.58	1.34	1.21	1.18	1.26	1.43	1.23	1.70	1.71	1.09	1.59	1.64
20	1.43	1.62	1.25	1.62	1.31	1.46	1.31	1.36	1.20	1.12	1.26	1.69
